# Recent effective population size in Eastern European plain Russians correlates with the key historical events

**DOI:** 10.1038/s41598-020-66734-y

**Published:** 2020-06-16

**Authors:** Ural Yunusbaev, Arslan Ionusbaev, Giyoun Han, Hyung Wook Kwon

**Affiliations:** 1 Incheon National University, College of Life Science and Bioengineering, Incheon, 22012 South Korea; 2 Ufa Federal Research Center of the Russian Academy of Sciences, Institute of Biochemistry and Genetics, Ufa, 450054 Russia; 3 King Abdullah University of Science and Technology, Thuwal, 23955 Saudi Arabia

**Keywords:** Population genetics, Computational biology and bioinformatics, Genetics

## Abstract

Effective population size reflects the history of population growth, contraction, and structuring. When the effect of structuring is negligible, the inferred trajectory of the effective population size can be informative about the key events in the history of a population. We used the IBDNe and DoRIS approaches, which exploit the data on IBD sharing between genomes, to reconstruct the recent effective population size in two population datasets of Russians from Eastern European plain: (1) ethnic Russians sampled from the westernmost part of Russia; (2) ethnic Russians, Bashkirs, and Tatars sampled from the Volga-Ural region. In this way, we examined changes in effective population size among ethnic Russians that reside in their historical area at the West of the plain, and that expanded eastward to come into contact with the indigenous peoples at the East of the plain. We compared the inferred demographic trajectories of each ethnic group to written historical data related to demographic events such as migration, war, colonization, famine, establishment, and collapse of empires. According to IBDNe estimations, 200 generations (~6000 years) ago, the effective size of the ancestral populations of Russians, Bashkirs, and Tatars hovered around 3,000, 30,000, and 8,000 respectively. Then, the ethnic Russians exponentially grew with increasing rates for the last 115 generations and become the largest ethnic group of the plain. Russians do not show any drop in effective population size after the key historical conflicts, including the Mongol invasion. The only exception is a moderate drop in the 17th century, which is well known in Russian history as The Smuta. Our analyses suggest a more eventful recent population history for the two small ethnic groups that came into contact with ethnic Russians in the Volga-Ural region. We found that the effective population size of Bashkirs and Tatars started to decrease during the time of the Mongol invasion. Interestingly, there is an even stronger drop in the effective population size that coincides with the expansion of Russians to the East. Thus, 15–20 generations ago, i.e. in the 16–18th centuries in the trajectories of Bashkirs and Tatars, we observe the bottlenecks of four and twenty thousand, respectively. Our results on the recent effective population size correlate with the key events in the history of populations of the Eastern European plain and have importance for designing biomedical studies in the region.

## Introduction

Demographic events such as population expansion, contraction, and bottleneck are known to have a strong influence on the genetic variation in the individuals from the affected groups^1^. Therefore, the effective population size trajectory reconstruction is crucial for natural selection^2^ and genetic association studies^3^. Methods for a data-driven reconstruction of the recent^1,4–6^ and ancient^7–17^ effective population size have been extensively developed in the past decade. The recent effective population size in some European^5^ and American^6^ populations was studied, but in Eastern Europeans, it remains unclear.

**Figure 1:**
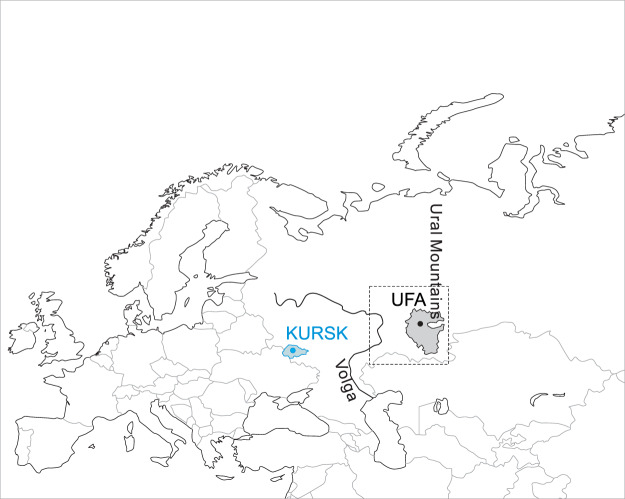
Source regions of the studied populations in the context of the Eurasian continent. KURSK and UFA (circles) are the capitals of the Kursk Region (KR, blue area) and the Republic of Bashkortostan (RB, grey area), respectively. The Volga-Ural Region (VUR, dashed block) is the area between the Volga River and the Ural Mountains. The figure created in CorelDRAW version 13.0.0.739 available at www.coreldraw.com.

In this study, we analyze the recent effective size of Russians from the Eastern European plain. For this, we use the previously published genome-wide datasets of GABRIEL^18,19^ consortium: the KURSK cohort sampled from the Kursk Region (KR) and the UFA cohort sampled from the Republic of Bashkortostan (RB, Table 1, Fig. 1). Patients and healthy controls of these cohorts geographically represent the western and eastern parts of the Eastern European plain (Fig. 1). Here we briefly describe their backgrounds relevant to our study. In the KR, 97% of the population comprises of ethnic Russians^20^. In the RB, the majority of the population currently represented by three ethnic groups: Russians, Bashkirs, and Tatars, each about 30% of the total population (Table 1). The ethnic Russians are Slavic-speaking people with genetic affinities to Central European populations^21^ that expanded to the East of the plain from their historical lands during the last several hundred years^22^. Bashkirs and Tatars are Turkic-speaking people indigenous to the Volga-Ural Region (VUR). Although Bashkirs and Tatars have cultural affinities to Turkic peoples from Central Asia, their genetic makeup is predominantly of European ancestry with varying proportions of genetic contribution from South Siberian and Central Asian populations^23,24^. Although the considered ethnic groups reside together in the VUR, they are relatively isolated due to the linguistic and religious differences that are likely to hinder the gene flow^25^.

**Table 1:** Comparison of study populations. *Official website of the Russian Census^20^.

Ethnic group	Kursk region				Republic of Bashkortostan			
	KURSK cohort		Census*		UFA cohort		Census	
	n	%	n	%	N	%	n	%
Russians	541	100	1,036,561	97	285	42	1,432,906	35
Bashkirs	—	—	—	—	159	24	1,172,287	29
Tatars	—	—	1,279	0.1	229	34	1,009,295	25
Others	—	—	89,241	2.9	—	—	457,804	11
Total	541	100	1,127,081	100	673	100	4,072,292	100

Thus, we investigated two cohorts sampled from the area of origin of ethnic Russians (KURSK), and from the area, where the ethnic Russians migrated and came into contact with indigenous peoples (UFA). These cohorts provide a good opportunity to study the recent effective population size in two complex models: (1) a population that exponentially grows and expands to a vast new area while coming in contact with different small populations; (2) small indigenous populations interact with each other while the third big population of migrants comes into contact with them and exponentially grows. These models were recently studied in Americas^6^, which were isolated from their source populations left in another continent. The ethnic Russian migrants in our study are not isolated from their source population. In addition, the recent history of the considered ethnic groups is well studied, which allows us to compare the data-driven demographic reconstructions to the key historical events.

We used the IBDNe^5^ approach to estimate the recent effective population size. Then to check the accuracy of the IBDNe estimated demography trajectories, we compared them with the DoRIS^1^ optimized demography scenarios (see details in the section “Methods”).

Here we ask whether the genomic-data-driven reconstruction of the recent effective size in Russians fits the key historical events such as migration, war, colonization, famine, establishment, and collapse of empires. Specifically, we ask whether it is possible to distinguish the ethnos-specific signals in the recent demography trajectories revealed from the genomes of distinct ethnic groups of the mixed dataset and whether these signals fit the corresponding ethnos-specific key historical events.

## Results

### Effective population size estimation by IBDNe

**Figure 2:**
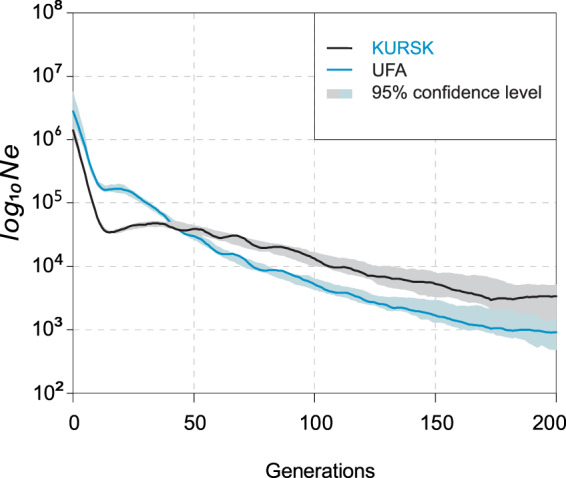
The overall recent effective population size of the KURSK and UFA cohort.

First, we compared the IBDNe-estimated (see details in the section “Methods”) overall effective population size in the KURSK and UFA cohorts (Fig. 2). The UFA cohort had a lower effective size for the first 50 generations before the present, even though it had a larger size in the earlier generations (Fig. 2). This disagreement suggests that the ancestors of these cohorts were drawn from different source populations. Taking into account that the UFA cohort includes individuals of different ethnic backgrounds, it is interesting to ask whether its trajectory fits the true history of each ethnic group. To this extent, the three datasets of Bashkirs, Russians, and Tatars have been separated out from the UFA cohort according to self-reported data of the individuals.

**Figure 3:**
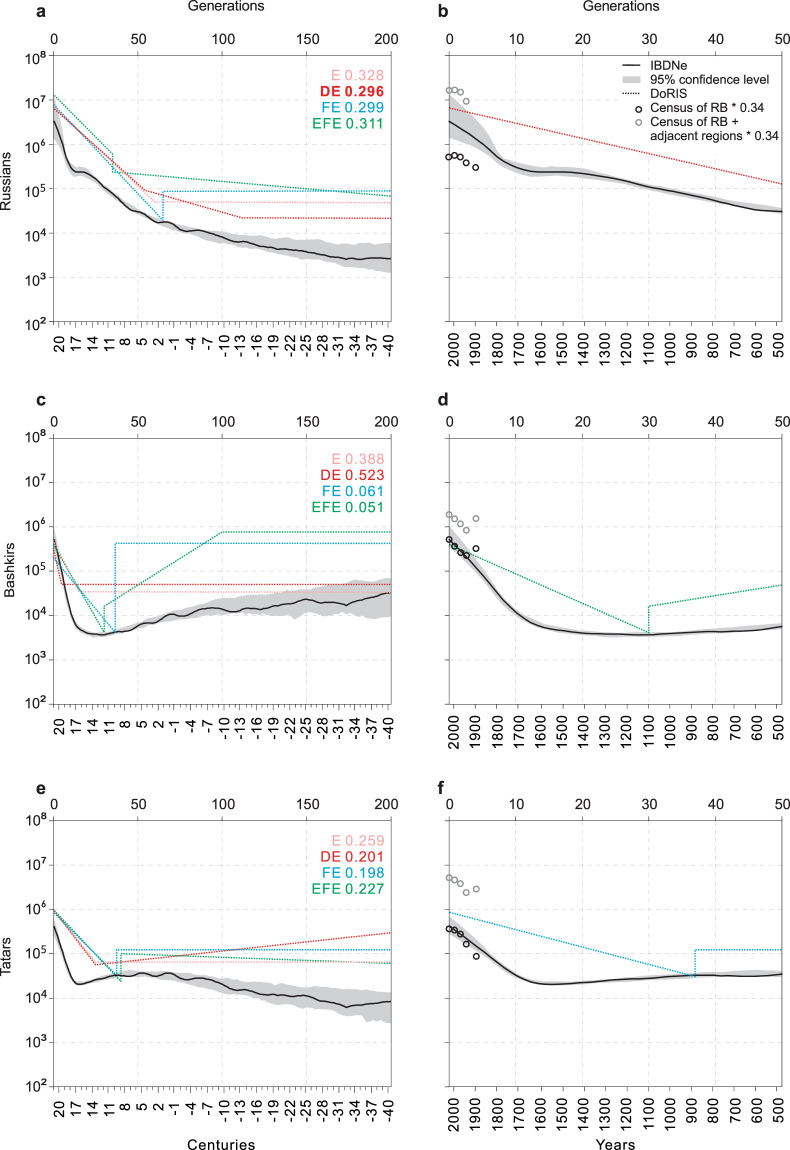
The recent effective population size of the ethnic groups of the UFA cohort. The left panel shows the estimated population size trajectories for the past 200 generations, and the right panel shows the more detailed zoomed-in trajectories for the past 50 generations (assuming 30 years per generation). DoRIS demographic scenarios: (E) exponential expansion (or contraction); (DE) double exponential expansion; (FE) founder event followed by exponential expansion; (EFE) expansion (or contraction) followed by founder event, then exponential expansion. Each scenario is accompanied by the corresponding optimal RMSE. The right panels show the census of the RB (black circles) and the census size of the RB + adjacent regions (grey circles).

We separately assessed the population size for each ethnic group using the IBDNe. The results of this assessment are reported in Fig. 3 and Tables S3.1-S3.3. The results suggest that historical demographic trajectories in studied populations are surprisingly different. The left three panels of Fig. 3 show the effective size trajectories estimated by the IBDNe in the three ethnic groups for the last 200 generations. The IBDNe estimated effective size in the Russian ethnic group shows constant growth with increasing rates from 3,000 to 3,300,000 (Fig. 3a). The IBDNe estimated trajectories of the ethnic Russians from the UFA (Fig. 3a) and KURSK (Fig. 2) cohorts agree. It shows how the considering of the hidden structure of the sample improves the accuracy of the effective size estimation. In contrast, Bashkirs decreased from 33,000 until 4,000, and then approximately 15 generations ago, they began to grow at increasing rates. In Bashkirs, the bottom of the IBDNe curve was about 30 generations ago. In Tatars, the IBDNe shows the increasing trajectory from 8,000 to 408,000, with the short-time drop until 20,000, which was about 15 generations ago. In Fig. 3, it was observed that the indigenous Bashkirs and Tatars experienced the bottleneck in contrast to the ethnic Russians. Especially, the Bashkirs experienced an extended period of low population size between the 15th and the 30th generations. These trajectories are well fit with the written historical data, which is described in the section “Comparison with historical records”.

Next, we asked whether the population size trajectories inferred by the IBDNe in the ethnic groups are accurate and reliable. To check this, we compared the IBDNe revealed population size trajectories with the DoRIS optimized demographic models, written historical data, and census.

### Evaluation of IBDNe inference comparing to DoRIS demographic scenarios

We evaluated the accuracy of IBDNe inferred demography trajectories via comparing them to corresponding inferences of the DoRIS approach. For this, we optimized the demographic scenarios for each ethnic group of the UFA cohort using the DoRIS approach (Fig. 3, see details in the section “Methods”). The following demographic scenarios were tested by the DoRIS: (E) exponential expansion (or contraction); (DE) double exponential expansion, when population size grows with increasing rates; (FE) founder event followed by exponential expansion; (EFE) expansion (or contraction) followed by founder event, then exponential expansion. The abbreviations of the tested demographic scenarios and the corresponding optimal root mean square error (RMSE) are shown at the top right of each left panel of Fig. 3. Based on the smallest value of the RMSE, the following three models were selected for the studied populations: DE for Russians, EFE for Bashkirs, FE for Tatars.

According to the DoRIS optimized scenario, the population of the ethnic Russians was of constant size before the 115th generation (~13th century BC, assuming 30 years per generation), then it was growing until the generation 65 (~5th century AD), after that it began to grow with increasing rates. In IBDNe trajectory, detailed population size is not observable because the curve is smoothed by averaging the data from multiple random starts^5^. In any case, Fig. 3a shows that in the Russian ethnic group, the DoRIS demography model with the optimal RMSE agrees with the IBDNe revealed demography trajectory.

The remarkable aspect of this analysis is the more accurate timing of the most notable demographic events. For example, in Bashkirs, we can see that the most dramatic population loss occurred in the 30th generation that approximately fits the 12th century. Interestingly, DoRIS and IBDNe agreeably show that Bashkirs originate from the ancestral population of a greater effective size, which existed for more than 200 generations (~6000 years) ago. Both methods show that the ancestral population of Bashkirs contracted during the period from 100 to 30 generations ago.

In Tatars, according to the FE model with the lowest value of the RMSE, the founder effect-like event occurred in generation 37 (Fig. 3f), which overlaps with the 8th century. The IBDNe also shows the population contraction at that period. The RMSE optimization in the FE model suggests that before the 37th generation, the ancestral population of Tatars was of constant size. Here we mention that the FE model does not show the exact number of generations that existed before the founder effect, which raises the uncertainty in the period before the bottleneck. The IBDNe shows that the effective size of Tatars increased before the 37th generation. Due to the above-mentioned uncertainty and disagreement in data revealed by different methods, the demography trajectory of Tatars before the 37th generation remains unclear.

In sum, the results suggest that the IBDNe trajectories in the studied populations agree with the corresponding demographic scenarios inferred by the DoRIS.

### Comparison with historical records

#### Russians

The ethnic Russians formed from East Slavic tribes native to European Russia with cultural ancestry based in Kievan Rus^26,27^. During the 6th to 7th centuries, Slavs commenced exploring the East European Plain widely^28^. The IBDNe and DoRIS data show that approximately after the 5th century onward, the population of ethnic Russians began to grow with increasing rates (Fig. 3a).

According to the traditional viewpoint, the Mongol invasion in the 13th century affected the Rus principalities dramatically^26,27^. In contrast to the traditional viewpoint, Gumilev (1989)^29^ argued that Mongols played a positive role in medieval Russia. Interestingly, the IBDNe and DoRIS estimations both support the Gumilev’s (1989)^29^ opinion. Figure 3a,b show that the effective size of the Russian ethnic group keeps growing with increasing rates in the 13th century.

Another remarkable demographic event in the ethnic Russians’ IBDNe trajectory is a slowdown of the growing rates 16 generations ago (Fig. 3a,b) that overlaps with the Time of Troubles in the 17th century well known in Russian history as The Smuta. At that time, according to historical records, a deep social and economic crisis accompanied by drought, plague, Polish, and Swedish interventions led to the decrease of Russian peasant’s number by 4 times^27,30,31^.

#### Bashkirs

In the 10th century, Persian chroniclers mentioned that “Bashkirs were independent tribes occupying lands on the Ural, Volga, Kama, and Tobol Rivers”. According to historical^32–34^ and genomic data^24^, the autochthonic Uralic Bashkir tribes admixed Turkic tribes migrated from South Siberia (Fig. S1) in the 11–13th centuries. Interestingly, the estimated trajectories in Fig. 3c suggest that the effective size of the ancestral population of Bashkirs existed about 200 generations (~6000 years) ago was rather big, hovering around 30,000 and reaching up to 700,000 according to IBDNe estimation, and up to 950,000 according to DoRIS estimation. Then, the population size dramatically contracted. The earliest written historical data on Bashkirs’ population size refers to the 10th century, and it agrees with our population size assessments. For example, the IBDNe and DoRIS data suggest that the effective population size of Bashkirs’ in the 10th century hovered from 5,000 to 10,000. Ibn Fadlan, in 922, wrote that “Bashkirs are warlike and powerful people with 5,000 militaries”^35^.

The notable demographic event detected by the DoRIS is the drop in population size in Bashkirs 30 generations ago, approximately in the 12th century. According to written historical data in 1220, Mongols invaded Bashkirs’ lands around the South Urals. There are two opinions on Bashkirs’ history in the Mongol invasion period. The first opinion is that Bashkirs resisted Mongols and won multiple battles in 1220–1236 (Fig. S1), which cost them a high human loss. Then a treaty of friendship and alliance between Bashkirs and Mongols was concluded^29,36–39^. Another opinion^34,40^ is that the bulk of Bashkir tribes benefited from voluntarily joining the Mongol Empire. The IBDNe and DoRIS both show a historical minimum of the Bashkir population in the ~12th century that supports the first opinion.

After the Golden Horde had broken apart, the territory of the modern RB was divided between the khanates of Kazan and Sibir until the Russians conquered the Kazan in the 16th century. It is widely assumed that Bashkirs benefited after joining the Russian Empire of their own volition in the 16th century^33,34,41^. The alternative opinion is that Bashkortostan did not join the Russian Empire of its own volition, but was conquered in 16–17th centuries^42–46^. In this period, in Bashkirs, the IBDNe assessed a prolonged drop in the effective population size. To clarify this phenomenon, we carefully examined the historical records of that period.

The story of Russian Asiatic extension started around 1552 with the annexation of Kazan and subsequent extension down the Volga. It required two centuries and three extensions (in 1652–57, the 1730s, and 1740s) of the frontier defense lines beyond the Volga^42–46^. The last one was the Orenburg defense line on the lands of Bashkir tribes. Bashkirs resisted Russian advances in a series of frontier wars and minor border outbreaks in 1572, 1581, 1586–87, 1610–13, 1662–64, 1675–83, 1705–11, and 1735–40^44^. According to Donnelly’s (1968)^44^ estimates, the last decisive war (Fig. S1) cost Bashkirs about a third of the 100,000 population. According to the official report of the Orenburg expedition in 1735–40, Bashkirs lost about 60,000 individuals^42^. These historical records agree with our recent population size estimations. The IBDNe data (Fig. 3a) shows that the Bashkir population in the 16–17th centuries did not recover.

Bashkirs were pacified by the end of the 18th century. In 1798 the Spiritual Assembly of Russian Muslims in Ufa city was established, an indication that the imperial government recognized the rights of Bashkirs. In 1919, the Ufa Governorate became the RB as a part of the Russian Federation. The IBDNe and DoRIS data are consistent that in the 18th century, the effective population size of Bashkirs started to grow exponentially (Fig. 3d).

#### Tatars

Contemporary Volga Tatars originate from Volga Bulgars, which were Turkic tribes^47^, who settled north of the Black Sea and founded Old Great Bulgaria (Fig. S1) in the 7th century. In this period, the IBDNe showed that the effective population size of Tatars reached the first peak before contraction and hovered around 40,000. According to the DoRIS data, the effective population size in Tatars at that time was about 100,000. It is bigger than in Russians and Bashkirs in the same period. Then in the 8th century, the part of Bulgars split and migrated to the VUR^48^. At this period in Tatars, the DoRIS indicates the most significant population drop from 100,000 to 25,000. Perhaps, the DoRIS identified the split as a loss of the bulk of the population. In the 11th century, Bulgarian cities were several times devastated by Russian principalities of Novgorod and Vladimir^49^. The IBDNe in this period shows a population contraction.

In 1223–1236, Mongols occupied the South of Volga Bulgaria^36,37^ and the part of Bashkirs’ lands. At this, period the IBDNe shows (Fig. 3f) a contraction of Tatars’ population size, but it was not replicated by the DoRIS. Meanwhile in Fig. 3d, the DoRIS, and IBDNe together show the strong signals of dramatic population loss in Bashkirs. Hence, we can suppose that the Mongol invasion mostly devastated Bashkirs, which were the first in Mongols’ way to Bulgaria in 1236 (Fig. S1). In the 13th century, Volga Bulgaria became a part of the Golden Horde with the capital in Kazan. After the disintegration of the Golden Horde in the 15th century, Volga Bulgaria became an independent khanate^50–52^. In 1552, Ivan the Terrible captured Kazan (Fig. S1) after a long siege, and the khanate was subjugated to Russia^53^. At this time, which is around the 15th generation, the IBDNe curve reaches the bottom near point 20,000 (Fig. 3f). In the 18th century, trading and industry greatly developed the Kazan Governorate of the Russian Empire. At that time, according to IBDNe data, Tatars’ population began to grow exponentially. In 1920 Kazan Governorate became the Republic of Tatarstan of the Russian Federation^48^.

There are other major historical events that should have been reflected in the recent demography of Russians. For example, The Russian Civil War in 1918–1922 and The Great Patriotic War in 1941–1945. In Tatars and Russians, the IBDNe trajectory and census grew with increasing rates in both wars. In Bashkirs, after The Russian Civil War, the census data dropped. However, this event has not reflected in the IBDNe results. Our data suggest that the wars of the 20th century affected the total population size of Russians, but not the effective population size.

### Comparison with census data

According to the census data^20^ and the distribution map of populations (Fig. S1), the UFA cohort might represent a random sample of Bashkirs and Tatars from the VUR. Nevertheless, it is unclear whether the UFA cohort is enough to assess the effective population size in each studied ethnic group from the VUR. The UFA cohort was sampled from the RB, but the part of Bashkirs and Tatars live in adjacent regions of the RB (Fig. S1, Table S1.1-S1.3). To this extent, we first compared the IBDNe estimation with the census data from the RB and then with the summarised census data from the RB and its adjacent regions. The right panels of Fig. 3 show the census of the RB (black circles) and the summarized census size of the RB and its adjacent regions (grey circles). In our study, we used the data available from the census 2010, 1979, 1959, 1926, 1897. We let the *g* = 0 generation corresponds to the year in which the average age of the sample was 30, that is, in 2010. We assumed a 30-year generation time. Consequently, the *g* = 1 generation corresponded to 1979. Figure 3 and and Tables S1.1-S1.5 shows the available census data which approximately corresponds to *g* from 0 to 4 as follows: 2010 (*g* = 0); 1979 (*g* = 1); 1959 (*g* = 2); 1926 (*g* = 3); 1897 (*g* = 4).

The effective population size is expected to be several times smaller than the census size, because of the latter one includes elderly individuals and children^54^. Previously, it was suggested that in the modern human population, a ratio of effective size to census size is around 0.34^55^. In Fig. 3d, black circles representing 34% of census well overlap with the confidence intervals of the IBDNe estimated effective population size trajectories in Bashkirs. It suggests that the sample of ~160 individuals is enough to assess the recent population history when the current overall size is ~1,000,000. However, the census size of Bashkirs in 1897 was larger than the confidence interval and then dropped in the census 1926. This shift is consistent with the data on population loss in the Russian famine of 1921–22, which primarily affected the VUR^56–58^. In Tatars, the IBDNe estimated effective size matches the census data for the generations 1 and 2, but it is too high for the generation 0. We suppose that the extrapolation of earlier growth rates overestimated the size of the generation the 0. Tatars’ population growth rate dropped between 1897 and 1926. It is likely the famine of 1921–22 and migration affected the growth rates for the generations 3–4 in Tatars.

**Figure 4:**
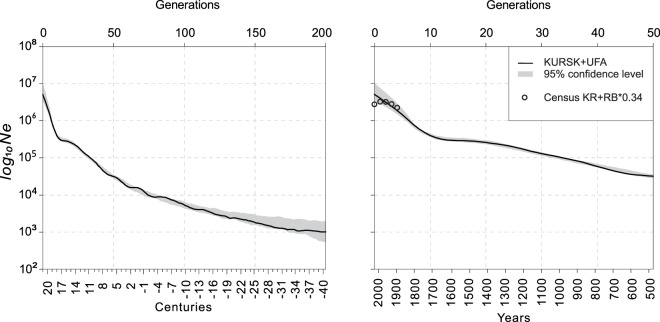
The recent effective population size of the ethnic Russians in the merged dataset from the KURSK and UFA cohorts.

We show (Fig. 3d,f) that in small indigenous subpopulations, the IBDNe estimations of the recent effective size well fits with the RB census data. However, in ethnic Russians, the IBDNe overestimates the recent effective size (Fig. 3b), suggesting that the ethnic group of Russians from the UFA cohort is not sufficiently representative of the ethnic Russians’ population to give reasonable estimates. Taking into account that ethnic Russians in the UFA cohort comprised of migrants from Western Russia, we speculated that the ethnic Russians from the KURSK and UFA cohorts combined into one dataset should better represent the recent demography history of the ethnic Russians. To this extent, we merged ethnic Russians from the KURSK and UFA cohorts and calculated their recent effective size for the merged dataset. Next, we summarised the number of ethnic Russians from the census of the KR and RB (Table 1.3–1.5. The result is shown in Fig. 4. In the merged dataset of ethnic Russians, the IBDNe estimated effective size overlaps with the census data. The only exception is the generation 0 overestimated due to the extrapolation of earlier high growth rates.

The remarkable aspect of the trajectory of the ethnic Russians is the high level of growth rate in the last 50 generations. To this extent, the relatively short-term slowdown of growth rate near generation 15 is intriguing. Previously it was shown that the overrepresentation of short (<6 centimorgans, cM) IBD segments might lead to oscillation in the recent generations^5^. Here we ask whether the oscillation between the generations 10–20 in the Russian ethnic group is a real demography history or an artificial signal. To reduce the oscillation in the recent generations, we recalculated the effective size trajectory in ethnic Russians using the IBD segments longer than 6 cM. In addition, the dataset used in the analysis was doubled by merging the KURSK and UFA cohorts. The results are shown in Fig. 4. The ethnic Russians’ trajectories from Figs. 3a,b, and 4 show that increasing the dataset size from 285 to 826 and the IBD segment length threshold from 4 to 6 cM resulted in oscillation reduction. However, in ethnic Russians, the population size drop near the 15th generation is still distinguishable. It gives the ground to assume that this drop is a true signal, which reflects the population loss on The Smuta^27^ in the 17th century.

The similarity of the estimated demographic histories of the ethnic Russians from the KURSK and UFA cohorts suggests that these two subpopulations have a shared demographic history. In particular, the similarity of the estimated demographic histories before the 10th generation agrees with previous data^22^ that most of the ethnic Russians in the VUR population originated from Western Russia.

## Discussion

Our study revealed the recent effective population size trajectories in Russians from the Eastern European plain. Here we discuss whether the inferred trajectories and approximate timescales in Fig. 3 fit the key historical events. The inferred trajectory of the ethnic Russians starts to grow exponentially with increasing rates after the 115th generation, which overlaps with the 5th century in the approximate timescale of Fig. 3a. According to historical records, Slavs commenced exploring the East European Plain in 6th century^28^. The Russian ethnic group’s trajectory shows a moderate drop about 16 generations ago that overlaps with the 17th century of the approximate timescale and fits the historical records on Time of Troubles in the 17th century well known in Russian history as The Smuta^27^. We estimate that the effective population sizes in Bashkirs and Tatars decrease in the generations after the Mongol invasion and after the commencement of Russian expansion to the East reaching a minimum around 15–20 generations ago, but recovering within the last 10 generations. Bashkirs have the smallest bottleneck size of four thousand, while Tatars have a greater bottleneck size of twenty thousand. Historical records prove substantial human loss in Bashkirs in conflicts of 16–18th centuries^42,44^. Approximate timing of the contractions and bottlenecks in the trajectories of Bashkirs and Tatars (Fig. 3c,e) agree with the timetable of the historical records on conflicts that affected these two populations in the 16–18th centuries. The approximate timing of the first pick of the Tatar’s trajectory overlaps with the time of the establishment of Old Great Bulgaria in the 6th century, which is well known from historical records^47,48^. This data gives ground to conclude that the IBDNe-inferred demography trajectories fit the key historical events of considered ethnic groups.

We can also look at the duration of the founder effect that the indigenous populations experienced, bearing in mind the historical events discussed above. Bashkirs experienced a prolonged severe founder effect that presumably initiated by the Mongol invasion in the 13th century and extended until the 17th century due to the series of conflicts^43,44^ during Russians’ expansion to the East. In Tatars and Bashkirs, we observe the two different patterns of a bottleneck related to the duration and severity of the population drop. In Tatars, the drop in size was of short-time presumably related to founder effect induced by the negative impact of the short war^53^ in 1552 resulted in Kazan Khaganate fall where Tatars are originated from. The ethnic Russians did not experience the founder effect at the considered time.

Another remarkable aspect of our inference is the high degree of concordance between the estimates from the Tatars’ and Bashkirs’ datasets 150–200 generations ago. Both populations contracted during the 175–200th generations (~32–40th centuries BC). Notably, the contraction rate in Bashkirs was higher. Then both populations grew until the generation 150 (~25th century BC). After the 150th generation, the demography trajectory of Bashkirs showed a sharp pick and started to drop. At the same time, the trajectory of Tatars smoothly went to the plateau. Meantime, 200 generations ago, the ancestral population of Bashkirs was several times bigger than in Tatars. These findings suggest that the ancestral populations of Bashkirs and Tatars were divergent but affected by the same factors. Interestingly, during the 175–200th generations, the number of ethnic Russians was of constant size and lower than in Bashkirs and Tatars.

Here we split the multiethnic cohort to ethnic subpopulations that led to a decrease in the dataset size. Hence, the next question that needs to be discussed whether it might affect the accuracy of the effective size estimations. To this extent, we tested the applicability of the IBDNe approach in the small ethnic subpopulations of a few hundred individuals and compared it with the census data. The concordance between these estimates and corresponding census data is excellent. However, the important caveat revealed from our case study is that the IBDNe estimations using a small dataset reflect the recent demography of only the part of the population included in the territory where the studied cohort was sampled. These estimations cannot be extrapolated to the adjacent territories. For example, in Bashkirs and Tatars sampled from the territory of the RB estimated recent effective size well fits with the census of the RB and does not agree with corresponding census data of territories adjacent to the RB (Fig. 3d,f). This finding suggests that the recent gene flow between Bashkirs and Tatars living out of the RB is low. In the ethnic Russians, we also found that the IBDNe estimated recent effective size fits with the census data from the distinct region where the cohort was sampled. In the ethnic Russians, the best concordance between the estimated effective size and the census data was obtained from the merged dataset, which comprises the individuals from the KURSK and UFA cohorts.

In conclusion, our results provide detailed information on the recent effective size in populations of Eastern European plain, including approximate timing of bottlenecks and have importance for designing biomedical studies in the region. In addition, our case study demonstrates the applicability of IBD-haplotype based methods to estimate the recent effective size in a mixed population. Specifically, we show that these methods are powered to distinguish ethnos-specific recent demography signals in a few hundred genomes subset of the multiethnic cohort.

## Methods

### Samples and quality control

We used Illumina 650k array genotyped genomes of 1238 individuals (610 asthma patients and 628 healthy controls) generated by the GABRIEL Consortium^18,59^. Quality control was done as in Yunusbayev *et al*. (2015)^24^, where authors used IBD distribution to infer the recent demographic history. Individuals with more than 1.5% missing genotypes were removed from the dataset. Only markers with a 97% genotyping rate and minor allele frequency (MAF) > 1% were retained. The absence of cryptic relatedness corresponding to first- and second-degree relatives in our dataset was confirmed using the KING software^60^. After filtering the genotypes for quality, we explored genetic distances between Linkage Disequilibrium (LD) pruned genomes using the MDS plot and removed outliers (Fig. S2). The filtering steps resulted in a dataset of 1214 individuals (600 asthma patients and 614 healthy controls) and 486,561 SNPs available for the downstream analyses. Genetic distances between SNPs in centimorgans were incorporated from the GrCh37 genetic map generated by the HapMap project^61^. In Fig. S2 patients are uniformly distributed among the healthy controls randomly sampled from the population. It gives the ground to consider that the disease markers do not disturb the sharing of IBD haplotypes used for the inference of the effective population size.

### IBD segments detection

We used the IBDSeq version r1206 software^62^ with the default parameters except minibd = 2 to detect chromosomal tracts that are IBD between pairs of individuals. We used the HapMap recombination map^61^ to calculate the genetic distances. Altogether, we detected 7 million IBD segments (Table S2) that were used to perform demographic inference in IBDNe and DoRIS analyses.

### Effective population size estimation

There are currently a few approaches powered to detect the signals of recent demographic events over the last 100 generations. We used the IBDNe^5^ version 04Sep15.e78 and the DoRIS^1^ version 03Nov17 algorithms. They were shown to have sufficient power to accurately estimate the recent effective population size in the genome-wide dataset of a few hundred individuals^1,4–6^. Both algorithms exploit the data on identical-by-descent (IBD) chromosomal segments sharing in a whole-genome dataset to infer the effective size of the ancestral populations. In a population, the IBD segments are co-inherited from common ancestors by pairs of individuals and delimited by historical recombination events. In the cohort that has been densely genotyped, the average detectable IBD segment is inherited from the common ancestor lived ten to a few hundred generations ago. Therefore, the information on IBD sharing is suitable to reveal recent demographic events.

We run the IBDNe with the following parameters minibd = 4 gmax = 200. First, we tested whether the SNP set used in our study is enough for reliable estimation of the effective population size using the IBDNe. Therefore, we created the dataset of 858 individuals from the 1958 British Birth Cohort (58 C) with SNP markers set used in our study. Then we estimated the effective population size trajectory for this dataset using the IBDNe (Fig. S3). We compared the British’s demography trajectories derived from the 58 C dataset and previously published^5^ WTCCC2 dataset. The concordance between the two estimates is excellent. In our study, we used datasets of different sizes (Table 1). To this extent, we mention that IBDNe was shown to distinguish different demography scenarios simulated in the datasets of 100, 200, and 1000 individuals^5^. The DoRIS can be used to infer the most likely demographic history patterns based on IBD sharing. For each dataset we run DoRIS with the following flags: “–DemographicModel Expansion”, “–DemographicModel DoubleExpansion”, “–DemographicModel FounderExpansion”, “–DemographicModel ExpansionFounderExpansion”.

Data analyses for this study were carried out in the Computational Cluster of Incheon National University Human Genome Research Center.

## Supplementary information

Supplementary Information.

Supplementary Information2.

Supplementary Information3.

Supplementary Information4.

## Data Availability

In this study, we analyzed the previously published dataset^18^ publicly available through the European Genome-phenome Archive (https://www.ebi.ac.uk/ega/home).
