# Supplementary material for: Recent effective population size in Eastern European plain Russians correlates with the key historical events
Source: Sci Rep. 2020 Jun 16;10:9729. doi: 10.1038/s41598-020-66734-y (PMC7298007; doi:10.1038/s41598-020-66734-y)
Supplement: Supplementary file 1 — Supplementary Information. [file 41598_2020_66734_MOESM1_ESM.pdf]

SUPPLEMENTARY INFORMATION

Recent effective population size in Eastern European plain Russians correlates with the key historical events

Ural Yunusbaev[1,2], Arslan Ionusbaev[3], Giyoun Han[1], Hyung Wook Kwon[1,*]

[1] Incheon National University, College of Life Science and Bioengineering, Incheon, 22012, South Korea

[2] Ufa Federal Research Center of the Russian Academy of Sciences, Institute of Biochemistry and Genetics, Ufa, 450054, Russia

[3] King Abdullah University of Science and Technology, Thuwal, 23955, Saudi Arabia

*Corresponding author hwkwon@inu.ac.kr

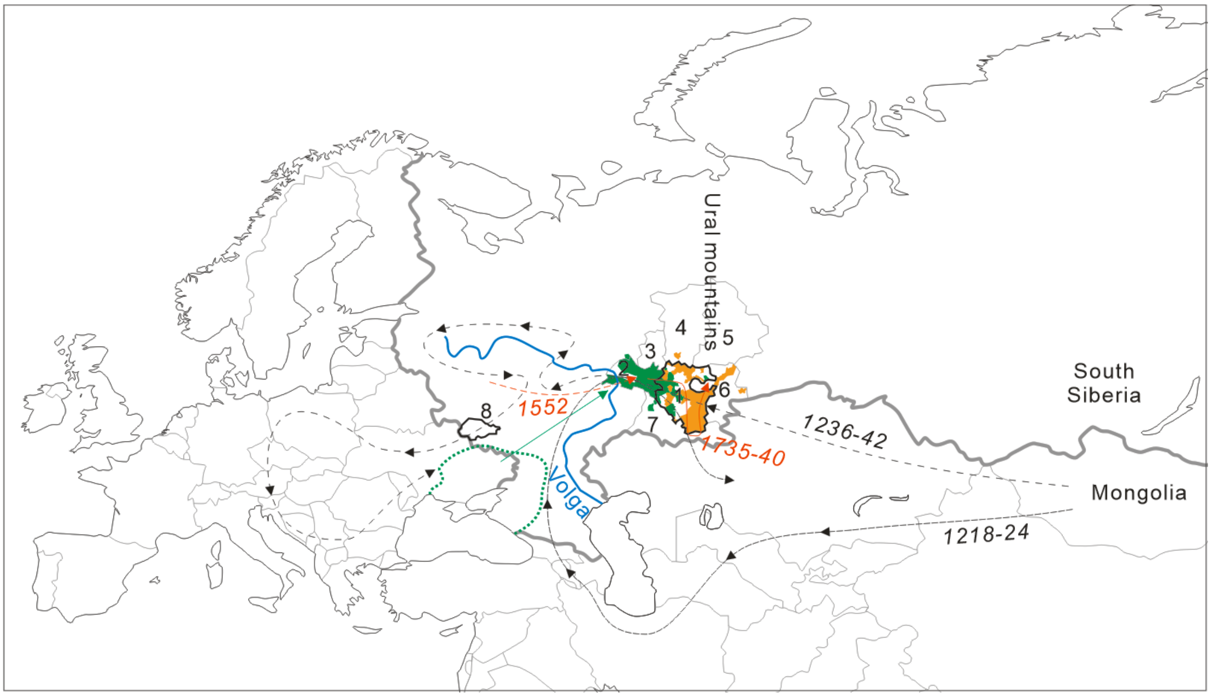

Figure S1. The current distribution of Bashkirs (orange) and Tatars (green) in the (1) Republic of Bashkortostan, (2) Tatarstan, (3) Udmurtia, (4) Perm, (5) Sverdlovsk, (6) Chelyabinsk, (7) Orenburg and (8) Kursk regions of the Russia Federation according to Census 2010 data[53] and most significant historical events affected the effective sizes of the studied populations: Mongol campaigns[32] (black arrows) in 1218-24 under the Subutai and Jebe, in 1236-42 under the Subutai; Russian campaigns (red arrows) in 1552 under the Ivan the Terrible[47], in 1735-40 under the Kirilov[37]; migration of Bulgars (green arrow) from Old Great Bulgaria (green boundaries) to the Volga in 8th century[45]. The figure created in CorelDRAW version 13.0.0.739 available at www.coreldraw.com.

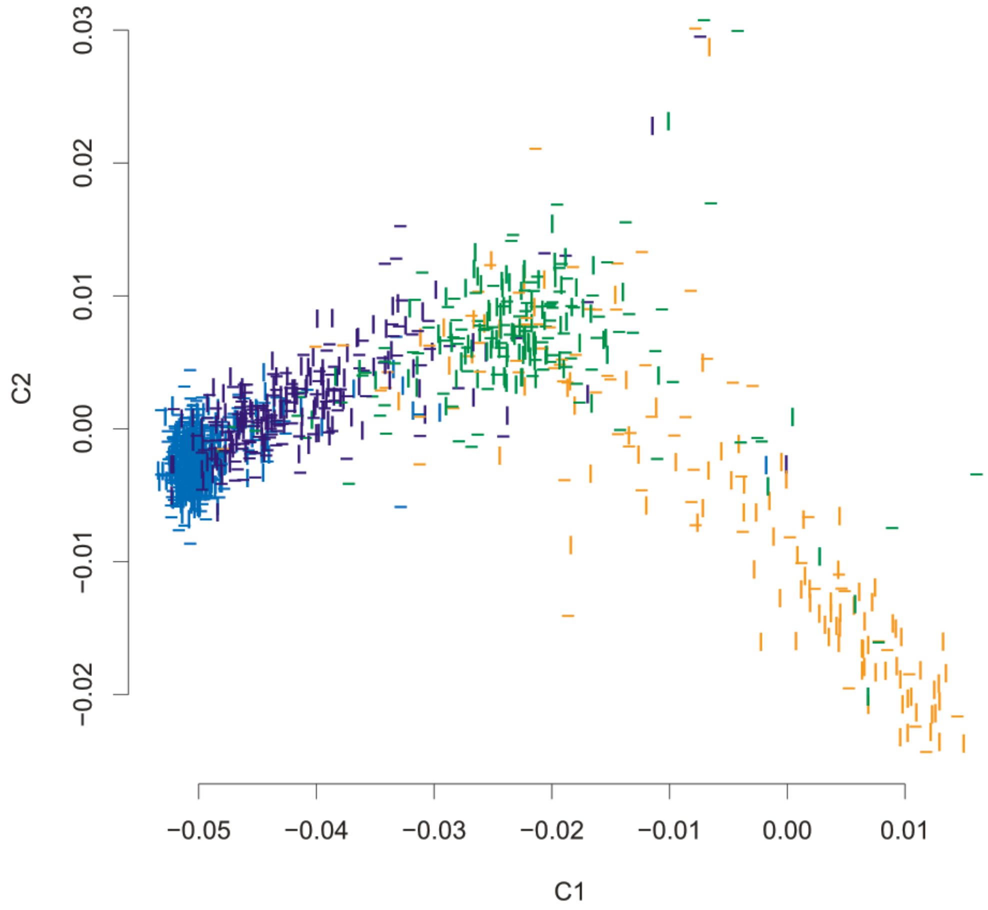

Figure S2. Multidimensional scaling of genome-wide identical-by-state distances for patients (dash) and controls (pipe) of the KURSK and UFA cohorts of the GABRIEL consortium. Ethnic groups of the UFA: Russians – dark blue; Bashkirs – orange; Tatars– green. The Russians of the KURSK – light blue.

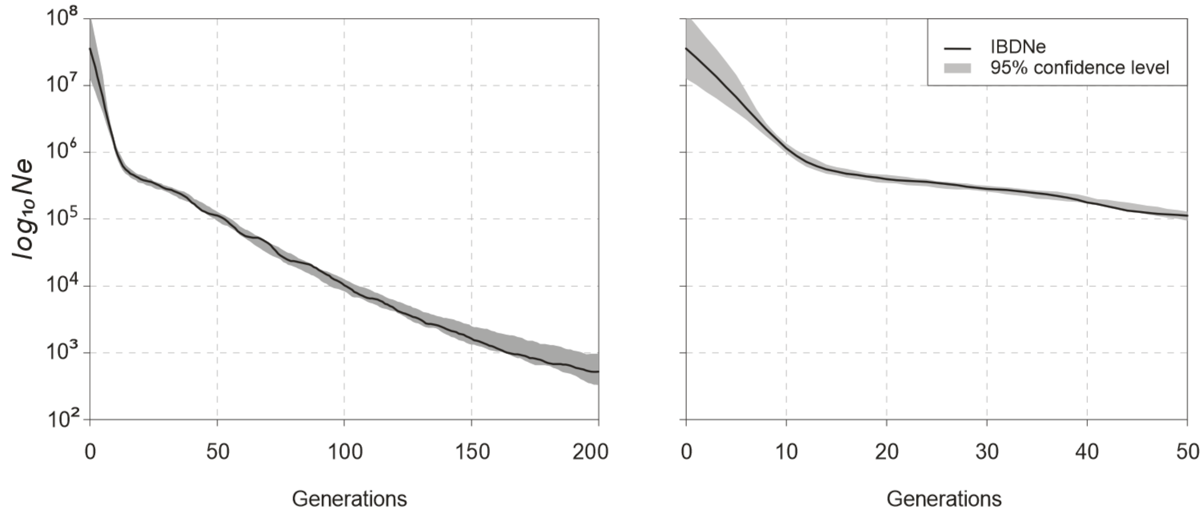

Figure S3. The recent effective population size estimated by IBDNe in the 1958 British Birth dataset with 486,561 SNPs used in our study.

Table S1.1. The population of the Republic of Bashkortostan (Census data).

Year (generation)	Russians	Bashkirs	Tatars
2010 (0)	1,432,906	1,172,287	1,009,295
1979 (1)	1,547,893	935,880	940,436
1959 (2)	1,418,147	737,711	768,566
1926 (3)	1,064,707	625,845	461,871
1897 (4)**	839,635	899,910	*245,729

* 245,729 includes 184,817 Tatars, 20,957 Mishars, 39,955 Tiptyars.

** Ufa Governorate Census 189.

Table S1.2. The population of the Bashkortostan and its adjacent regions (Census data).

Year (generation)	Russians	Bashkirs	Tatars
2010 (0)	14,072,504	1,462,589	3,682,487
1979 (1)	14,478,671	1,191,304	3,365,833
1959 (2)	13,078,272	913,738	2,820,913
1926 (3)	2,511,034	627,602	1,643,348
1897 (4)*	-	1,434,500	1,953,100

* The census of the European part of the Russian Empire in 1897.

Table S1.3. The population of the Bashkortostan and its adjacent regions (Census data).

Year	1926	1939	1959	1979	1989	2002	2010
**Republic of Bashkortostan**							
Russians	1,064,707	1,281,347	1,418,147	1,547,893	1,548,291	1,490,715	1,432,906
Tatars	461,871	777,230	768,566	940,436	1,120,702	990,702	1,009,295
Bashkirs	625,845	671,188	737,711	935,880	863,808	1,221,302	1,172,287
**Republic of Tatarstan**							
Russians	1,118,834	1,250,667	1,252,413	1,516,023	1,575,361	1,492,602	1,501,369
Tatars	1,164,342	1,421,514	1,345,195	1,641,603	1,765,404	1,981,356	1,982,609
Bashkirs	1,752	931	2,063	9,256	19,106	14,911	13,726
**Chelyabinsk region**							
Russians			2,372,164	2,650,836	2,929,507	2,965,885	2,829,899
Tatars			190,227	207,416	224,605	205,087	180,913
Bashkirs			88,480	124,825	161,169	166,372	162,513
**Orenburg region**							
Russians			1,296,458	1,432,450	1,568,442	1,611,509	1,519,525
Tatars			120,944	139,754	158,564	165,967	151,492
Bashkirs			30,126	41,733	53,339	52,685	46,696
**Sverdlovsk region**							
Russians			3,560,090	3,954,961	4,176,948	4,002,974	3,684,843
Tatars			158,222	179,341	183,781	168,163	143,803
Bashkirs			14,631	30,051	41,500	37,296	31,183
**Udmurt republic**							
Russians	327,493	679,294	758,770	870,270	945,216	944,108	912,539
Tatars	17,135	40,561	71,930	99,139	110,490	109,218	98,831
Bashkirs	5	362	1,150	3,608	5,217	4,320	3,454
**Perm region**							
Russians			2,420,230	2,506,238	2,592,246	2,401,659	2,191,423
Tatars			165,829	158,144	150,460	136,595	115,544
Bashkirs			39,577	45,951	52,326	40,740	32,730
**Total**							
Russians	2,511,034	3,211,308	13,078,272	14,478,671	15,336,011	14,909,452	14,072,504
Tatars	1,643,348	2,239,305	2,820,913	3,365,834	3,714,006	3,757,088	3,682,487
Bashkirs	627,602	672,481	913,738	1,191,304	1,196,465	1,537,626	1,462,589

Table S1.4. The population of the Kursk region (Census data).

Year (generation)	Russians
2010 (0)	1036561
1979 (1)	1361319
1959 (2)	1456350
1926 (3)*	1453180
1897 (4)*	1185506

*Census data from 1926 and 1897 were divided by 2 because, at that time, the Kursk Governorate was 2 times bigger than the contemporary Kursk Region.

Table S1.5. The **s**ummarised number of ethnic Russians from the census of the Kursk region and the Republic of Bashkortostan.

Year (generation)	Russians
2010 (0)	2469467
1979 (1)	2909212
1959 (2)	2874497
1926 (3)	2517887
1897 (4)	2025141

Table S2. The number of the IBDSeq detected IBD segments used for recent demography inference.

Ethnic group	Region	Cohort	IBD pairs, n
Bashkirs	RB	UFA	147,619
Tatars	RB	UFA	295,691
Russians	RB	UFA	464,061
Russians	KR	KURSK	1,836,979
Russians	RB + KR	UFA + KURSK	4,333,333
Total			7,077,683

IBDNe

Here we briefly describe input/output files and commands used in IBDNe software to infer recent effective population size for Bashkirs ethnic group. For details see http://faculty.washington.edu/browning/ibdne.html

For IBDNe estimations we used IBD pairs calculated by IBDSeq software (http://faculty.washington.edu/browning/ibdseq.html) as follows:

```
for i in `seq 1 22`; \
do java -Xmx64g -jar ibdseq.r1206.jar gt=bashkirs/chr.$i.vcf.gz \
out=bashkirs/ibdseq.$i.out nthreads=32; done
```

We started the IBDNe as follows

```
cat bashkirs/*.ibd | java -Xmx512g -jar ibdne.04Sep15.e78.jar \
map=grch37.map out=bashkirs/ne minibd=4 nthreads=32
```

IBDNe outputs are shown in Tables S2.1-S2.3

Table S3.1. The recent effective population size (*Ne*) with 95% confidential levels (*Cl*) estimated by IBDNe approach in Russian group of UFA cohort.
The table is in the separate file “Table S3.1.pdf”.

Table S3.2. The recent effective population size (*Ne*) with 95% confidential levels (*Cl*) estimated by IBDNe approach in Bashkir ethnic group of UFA cohort.
The table is in the separate file “Table S3.2.pdf”.

Table S3.3. The recent effective population size (*Ne*) with 95% confidential levels (*Cl*) estimated by IBDNe approach in Tatar ethnic group of UFA cohort.
The table is in the separate file “Table S3.3.pdf”.

DoRIS

Here we briefly describe input/output files and commands used in DoRIS software to infer the optimal demographic model for Bashkirs ethnic group. For details see https://github.com/pierpal/DoRIS

We started the DoRIS as follows:

```
java -jar DoRIS.jar \
--DemographicModel     ExpansionFounderExpansion \
--Grid                 OnePop.grid.ExpansionFounderExpansion.txt \
--AverageSharing       OnePop.Bashkirs.sharingDist.txt
```

Input file --Grid

```
cat OnePop.grid.ExpansionFounderExpansion.txt
current 250000 50000 500000
generation1 10 1 50
ancestral1 1000 1000 10000
```

```
ancestral2 20000 5000 50000
generation2 50 10 200
ancestral3 50000 100000 1000000
```

Input file --AverageSharing

#The average fraction of genome shared in Bashkirs

```
cat OnePop.Bashkirs.sharingDist.txt
1.    2.    0.00047246000000
2.    3.    0.00044335700000
3.    4.    0.00028643700000
4.    5.    0.00020101100000
5.    6.    0.00013887500000
6.    7.    0.00010385400000
7.    8.    0.00007155360000
8.    9.    0.00005112340000
9.    10.   0.00003495910000
10.   11.   0.00002657200000
11.   12.   0.00002104540000
12.   13.   0.00001465920000
13.   14.   0.00001045850000
14.   15.   0.00000770935000
15.   16.   0.00000656953000
```

Output file

```
tail -1 DoRIS.out
Done RMSE.      Best parameters found: Current: 300000.0
Generations1: 30.0      Ancestral1: 6000.0      Ancestral2: 20000.0
Generations2: 100.0     Ancestral3: 950000.0    error: 0.05143461619648559
```
