# Supplementary material for: Recent effective population size in Eastern European plain Russians correlates with the key historical events
Source: Sci Rep. 2020 Jun 16;10:9729. doi: 10.1038/s41598-020-66734-y (PMC7298007; doi:10.1038/s41598-020-66734-y)
Supplement: Supplementary file 2 — Supplementary Information2. [file 41598_2020_66734_MOESM2_ESM.pdf]

GEN	NE	LWR-95%CI	UPR-95%CI
0	3.30E6	1.40E6	1.34E7
1	2.67E6	1.25E6	9.84E6
2	2.13E6	1.10E6	7.16E6
3	1.68E6	9.69E5	5.14E6
4	1.31E6	8.40E5	3.60E6
5	1.00E6	6.62E5	2.40E6
6	7.44E5	5.45E5	1.48E6
7	5.34E5	3.84E5	7.28E5
8	4.19E5	3.05E5	4.83E5
9	3.46E5	2.62E5	4.33E5
10	2.96E5	2.27E5	3.89E5
11	2.66E5	2.01E5	3.59E5
12	2.47E5	1.85E5	3.33E5
13	2.40E5	1.85E5	3.17E5
14	2.38E5	1.89E5	3.00E5
15	2.40E5	1.95E5	3.18E5
16	2.40E5	1.99E5	3.11E5
17	2.37E5	1.91E5	3.10E5
18	2.32E5	1.88E5	3.04E5
19	2.26E5	1.79E5	2.95E5
20	2.17E5	1.70E5	2.79E5
21	2.05E5	1.64E5	2.56E5
22	1.95E5	1.57E5	2.34E5
23	1.84E5	1.49E5	2.12E5
24	1.73E5	1.41E5	1.92E5
25	1.61E5	1.34E5	1.76E5
26	1.48E5	1.25E5	1.64E5
27	1.35E5	1.15E5	1.50E5
28	1.23E5	1.05E5	1.38E5
29	1.13E5	9.63E4	1.28E5
30	1.05E5	8.91E4	1.20E5
31	9.90E4	8.31E4	1.14E5
32	9.23E4	7.64E4	1.06E5
33	8.63E4	7.13E4	9.82E4
34	8.05E4	6.72E4	9.20E4
35	7.53E4	6.31E4	8.55E4
36	6.94E4	5.90E4	8.05E4
37	6.48E4	5.49E4	7.57E4
38	5.96E4	5.12E4	7.09E4
39	5.43E4	4.84E4	6.59E4
40	4.92E4	4.55E4	6.04E4
41	4.58E4	4.32E4	5.55E4
42	4.21E4	4.03E4	5.21E4
43	3.85E4	3.74E4	4.97E4
44	3.58E4	3.45E4	4.78E4
45	3.37E4	3.30E4	4.49E4
46	3.24E4	3.16E4	4.31E4
47	3.15E4	3.01E4	4.10E4
48	3.10E4	2.84E4	3.87E4
49	3.05E4	2.73E4	3.63E4
50	3.00E4	2.62E4	3.44E4
51	2.92E4	2.51E4	3.22E4
52	2.81E4	2.39E4	3.09E4

53	2.72E4	2.32E4	2.98E4
54	2.56E4	2.26E4	2.86E4
55	2.41E4	2.15E4	2.72E4
56	2.25E4	2.04E4	2.61E4
57	2.11E4	1.95E4	2.49E4
58	1.95E4	1.86E4	2.39E4
59	1.86E4	1.78E4	2.32E4
60	1.78E4	1.70E4	2.21E4
61	1.72E4	1.63E4	2.17E4
62	1.72E4	1.55E4	2.08E4
63	1.73E4	1.50E4	2.04E4
64	1.74E4	1.43E4	2.00E4
65	1.76E4	1.40E4	1.96E4
66	1.78E4	1.36E4	1.91E4
67	1.77E4	1.31E4	1.84E4
68	1.73E4	1.26E4	1.82E4
69	1.69E4	1.21E4	1.76E4
70	1.61E4	1.17E4	1.71E4
71	1.53E4	1.15E4	1.65E4
72	1.42E4	1.13E4	1.60E4
73	1.33E4	1.10E4	1.56E4
74	1.23E4	1.06E4	1.55E4
75	1.18E4	1.03E4	1.52E4
76	1.15E4	1.01E4	1.48E4
77	1.12E4	9.89E3	1.46E4
78	1.10E4	9.65E3	1.43E4
79	1.09E4	9.74E3	1.40E4
80	1.11E4	9.69E3	1.36E4
81	1.12E4	9.38E3	1.35E4
82	1.13E4	9.16E3	1.34E4
83	1.14E4	8.87E3	1.32E4
84	1.16E4	8.66E3	1.29E4
85	1.15E4	8.36E3	1.26E4
86	1.15E4	8.08E3	1.23E4
87	1.14E4	7.67E3	1.20E4
88	1.12E4	7.30E3	1.18E4
89	1.09E4	7.10E3	1.16E4
90	1.05E4	6.86E3	1.13E4
91	1.03E4	6.75E3	1.14E4
92	1.00E4	6.62E3	1.13E4
93	9.80E3	6.47E3	1.12E4
94	9.49E3	6.37E3	1.12E4
95	9.14E3	6.20E3	1.12E4
96	8.82E3	6.06E3	1.11E4
97	8.67E3	5.90E3	1.10E4
98	8.45E3	5.75E3	1.10E4
99	8.21E3	5.73E3	1.08E4
100	7.86E3	5.83E3	1.06E4
101	7.62E3	5.86E3	1.06E4
102	7.37E3	5.82E3	1.04E4
103	7.13E3	5.56E3	1.01E4
104	6.83E3	5.57E3	9.87E3
105	6.66E3	5.57E3	9.60E3
106	6.48E3	5.34E3	9.53E3

107	6.33E3	5.35E3	9.46E3
108	6.31E3	4.95E3	9.26E3
109	6.27E3	4.83E3	9.07E3
110	6.30E3	4.75E3	8.96E3
111	6.36E3	4.67E3	8.91E3
112	6.41E3	4.66E3	9.01E3
113	6.39E3	4.67E3	9.03E3
114	6.31E3	4.70E3	8.95E3
115	6.15E3	4.51E3	8.76E3
116	5.90E3	4.46E3	8.59E3
117	5.82E3	4.39E3	8.56E3
118	5.67E3	4.43E3	8.64E3
119	5.51E3	4.45E3	8.72E3
120	5.30E3	4.43E3	8.69E3
121	5.14E3	4.35E3	8.81E3
122	4.98E3	4.29E3	8.82E3
123	4.94E3	4.09E3	8.80E3
124	4.94E3	4.04E3	8.54E3
125	4.89E3	3.87E3	8.30E3
126	4.80E3	3.74E3	8.09E3
127	4.74E3	3.72E3	7.86E3
128	4.76E3	3.71E3	7.61E3
129	4.72E3	3.60E3	7.50E3
130	4.63E3	3.55E3	7.41E3
131	4.48E3	3.46E3	7.32E3
132	4.28E3	3.37E3	7.50E3
133	4.28E3	3.32E3	7.62E3
134	4.36E3	3.27E3	7.71E3
135	4.38E3	3.22E3	7.80E3
136	4.38E3	3.17E3	7.94E3
137	4.36E3	3.12E3	7.92E3
138	4.31E3	3.07E3	7.98E3
139	4.23E3	3.08E3	7.84E3
140	4.17E3	2.91E3	7.47E3
141	4.11E3	2.93E3	7.26E3
142	4.08E3	2.80E3	7.03E3
143	4.10E3	2.78E3	6.83E3
144	4.07E3	2.71E3	6.91E3
145	4.00E3	2.61E3	6.94E3
146	3.96E3	2.50E3	7.00E3
147	3.94E3	2.52E3	7.01E3
148	3.86E3	2.54E3	6.96E3
149	3.81E3	2.61E3	7.06E3
150	3.69E3	2.58E3	7.13E3
151	3.60E3	2.57E3	7.15E3
152	3.59E3	2.54E3	7.19E3
153	3.57E3	2.52E3	7.15E3
154	3.52E3	2.54E3	7.12E3
155	3.43E3	2.54E3	7.23E3
156	3.36E3	2.53E3	7.19E3
157	3.28E3	2.51E3	7.07E3
158	3.24E3	2.45E3	6.91E3
159	3.22E3	2.35E3	6.71E3
160	3.17E3	2.26E3	6.38E3

161	3.11E3	2.25E3	6.23E3
162	3.03E3	2.23E3	6.06E3
163	2.97E3	2.20E3	5.95E3
164	2.91E3	2.17E3	5.89E3
165	2.91E3	2.14E3	5.81E3
166	2.86E3	2.10E3	5.72E3
167	2.84E3	2.04E3	5.56E3
168	2.82E3	1.94E3	5.48E3
169	2.80E3	1.87E3	5.64E3
170	2.76E3	1.80E3	5.75E3
171	2.70E3	1.72E3	5.81E3
172	2.64E3	1.70E3	5.78E3
173	2.54E3	1.68E3	5.75E3
174	2.62E3	1.70E3	5.68E3
175	2.67E3	1.71E3	5.60E3
176	2.69E3	1.69E3	5.49E3
177	2.68E3	1.69E3	5.44E3
178	2.65E3	1.67E3	5.47E3
179	2.60E3	1.65E3	5.66E3
180	2.54E3	1.63E3	5.85E3
181	2.55E3	1.62E3	5.95E3
182	2.53E3	1.63E3	5.93E3
183	2.59E3	1.61E3	5.91E3
184	2.63E3	1.58E3	5.96E3
185	2.67E3	1.52E3	6.03E3
186	2.68E3	1.47E3	5.99E3
187	2.72E3	1.53E3	5.89E3
188	2.75E3	1.51E3	5.69E3
189	2.74E3	1.46E3	5.45E3
190	2.74E3	1.40E3	5.50E3
191	2.72E3	1.39E3	5.62E3
192	2.73E3	1.37E3	5.71E3
193	2.73E3	1.36E3	5.76E3
194	2.73E3	1.34E3	5.80E3
195	2.70E3	1.32E3	5.87E3
196	2.66E3	1.30E3	5.92E3
197	2.65E3	1.27E3	5.96E3
198	2.64E3	1.27E3	5.94E3
199	2.65E3	1.28E3	5.93E3
200	2.66E3	1.28E3	5.91E3
