# Supplementary material for: Recent effective population size in Eastern European plain Russians correlates with the key historical events
Source: Sci Rep. 2020 Jun 16;10:9729. doi: 10.1038/s41598-020-66734-y (PMC7298007; doi:10.1038/s41598-020-66734-y)
Supplement: Supplementary file 3 — Supplementary Information3. [file 41598_2020_66734_MOESM3_ESM.pdf]

GEN	NE	LWR-95%CI	UPR-95%CI
0	5,19E+05	3,57E+05	1,05E+06
1	3,65E+05	2,59E+05	6,95E+05
2	2,55E+05	1,86E+05	4,54E+05
3	1,75E+05	1,32E+05	2,92E+05
4	1,19E+05	9,18E+04	1,84E+05
5	7,95E+04	6,20E+04	1,13E+05
6	5,20E+04	3,97E+04	6,87E+04
7	3,32E+04	2,47E+04	3,97E+04
8	2,23E+04	1,66E+04	2,53E+04
9	1,55E+04	1,23E+04	1,76E+04
10	1,13E+04	9,40E+03	1,32E+04
11	8,71E+03	7,50E+03	1,04E+04
12	7,00E+03	6,30E+03	8,87E+03
13	6,03E+03	5,33E+03	7,50E+03
14	5,38E+03	4,98E+03	6,49E+03
15	5,01E+03	4,49E+03	5,96E+03
16	4,71E+03	4,12E+03	5,60E+03
17	4,49E+03	3,98E+03	5,26E+03
18	4,29E+03	3,86E+03	4,93E+03
19	4,16E+03	3,71E+03	4,65E+03
20	4,01E+03	3,58E+03	4,53E+03
21	3,91E+03	3,52E+03	4,43E+03
22	3,87E+03	3,40E+03	4,29E+03
23	3,86E+03	3,24E+03	4,19E+03
24	3,85E+03	3,23E+03	4,11E+03
25	3,81E+03	3,25E+03	4,08E+03
26	3,75E+03	3,30E+03	4,06E+03
27	3,69E+03	3,34E+03	4,17E+03
28	3,66E+03	3,32E+03	4,21E+03
29	3,67E+03	3,31E+03	4,20E+03
30	3,70E+03	3,33E+03	4,22E+03
31	3,82E+03	3,34E+03	4,23E+03
32	3,90E+03	3,40E+03	4,21E+03
33	3,98E+03	3,43E+03	4,24E+03
34	4,06E+03	3,51E+03	4,34E+03
35	4,15E+03	3,64E+03	4,54E+03
36	4,19E+03	3,71E+03	4,65E+03
37	4,29E+03	3,83E+03	4,86E+03
38	4,34E+03	3,89E+03	5,01E+03
39	4,34E+03	3,95E+03	5,11E+03
40	4,31E+03	4,01E+03	5,26E+03
41	4,39E+03	4,00E+03	5,36E+03
42	4,43E+03	4,09E+03	5,47E+03
43	4,44E+03	4,26E+03	5,62E+03
44	4,51E+03	4,40E+03	5,86E+03
45	4,62E+03	4,49E+03	6,01E+03
46	4,82E+03	4,60E+03	6,19E+03
47	5,08E+03	4,68E+03	6,38E+03
48	5,39E+03	4,82E+03	6,56E+03
49	5,72E+03	4,95E+03	6,68E+03
50	6,05E+03	5,16E+03	6,87E+03
51	6,31E+03	5,22E+03	7,00E+03
52	6,51E+03	5,15E+03	7,21E+03

53	6,71E+03	5,30E+03	7,55E+03
54	6,74E+03	5,47E+03	8,01E+03
55	6,78E+03	5,72E+03	8,41E+03
56	6,79E+03	5,94E+03	8,72E+03
57	6,78E+03	6,13E+03	8,88E+03
58	6,69E+03	6,08E+03	8,83E+03
59	6,84E+03	6,12E+03	9,07E+03
60	6,99E+03	6,26E+03	9,40E+03
61	7,25E+03	6,58E+03	9,47E+03
62	7,68E+03	6,77E+03	9,70E+03
63	8,14E+03	7,01E+03	1,01E+04
64	8,61E+03	7,15E+03	1,03E+04
65	9,22E+03	7,23E+03	1,07E+04
66	9,82E+03	7,22E+03	1,10E+04
67	1,02E+04	7,36E+03	1,15E+04
68	1,05E+04	7,43E+03	1,18E+04
69	1,07E+04	7,51E+03	1,21E+04
70	1,07E+04	7,52E+03	1,22E+04
71	1,06E+04	7,72E+03	1,28E+04
72	1,03E+04	7,80E+03	1,30E+04
73	1,00E+04	7,99E+03	1,32E+04
74	9,75E+03	8,13E+03	1,33E+04
75	9,77E+03	8,23E+03	1,34E+04
76	9,76E+03	8,27E+03	1,36E+04
77	9,75E+03	8,27E+03	1,42E+04
78	9,95E+03	8,37E+03	1,46E+04
79	1,02E+04	8,41E+03	1,50E+04
80	1,06E+04	8,50E+03	1,54E+04
81	1,12E+04	8,68E+03	1,56E+04
82	1,16E+04	8,75E+03	1,59E+04
83	1,22E+04	8,74E+03	1,61E+04
84	1,27E+04	8,64E+03	1,66E+04
85	1,32E+04	8,84E+03	1,71E+04
86	1,36E+04	9,18E+03	1,77E+04
87	1,40E+04	9,41E+03	1,80E+04
88	1,41E+04	9,77E+03	1,81E+04
89	1,42E+04	1,00E+04	1,85E+04
90	1,44E+04	1,02E+04	1,90E+04
91	1,46E+04	1,02E+04	1,96E+04
92	1,48E+04	1,03E+04	2,03E+04
93	1,50E+04	1,01E+04	2,13E+04
94	1,49E+04	1,01E+04	2,21E+04
95	1,48E+04	1,01E+04	2,28E+04
96	1,46E+04	1,03E+04	2,33E+04
97	1,46E+04	1,06E+04	2,36E+04
98	1,43E+04	1,07E+04	2,39E+04
99	1,41E+04	1,05E+04	2,45E+04
100	1,37E+04	1,01E+04	2,47E+04
101	1,33E+04	1,04E+04	2,47E+04
102	1,30E+04	1,05E+04	2,49E+04
103	1,26E+04	1,07E+04	2,49E+04
104	1,22E+04	1,11E+04	2,50E+04
105	1,22E+04	1,14E+04	2,51E+04
106	1,21E+04	1,15E+04	2,50E+04

107	1,21E+04	1,15E+04	2,48E+04
108	1,23E+04	1,15E+04	2,53E+04
109	1,26E+04	1,11E+04	2,62E+04
110	1,30E+04	1,12E+04	2,67E+04
111	1,36E+04	1,15E+04	2,72E+04
112	1,41E+04	1,18E+04	2,78E+04
113	1,44E+04	1,12E+04	2,82E+04
114	1,46E+04	1,05E+04	2,84E+04
115	1,46E+04	1,11E+04	2,91E+04
116	1,43E+04	1,13E+04	2,94E+04
117	1,45E+04	1,11E+04	2,93E+04
118	1,45E+04	1,07E+04	2,95E+04
119	1,44E+04	1,10E+04	2,95E+04
120	1,41E+04	1,10E+04	2,93E+04
121	1,39E+04	1,07E+04	2,93E+04
122	1,37E+04	1,04E+04	2,98E+04
123	1,39E+04	1,03E+04	3,00E+04
124	1,43E+04	1,11E+04	2,93E+04
125	1,45E+04	1,10E+04	2,95E+04
126	1,47E+04	1,04E+04	2,95E+04
127	1,50E+04	1,03E+04	2,97E+04
128	1,56E+04	1,03E+04	3,04E+04
129	1,61E+04	1,02E+04	3,09E+04
130	1,64E+04	9,98E+03	3,12E+04
131	1,66E+04	9,71E+03	3,23E+04
132	1,65E+04	9,86E+03	3,29E+04
133	1,69E+04	9,95E+03	3,27E+04
134	1,74E+04	1,01E+04	3,25E+04
135	1,77E+04	9,97E+03	3,22E+04
136	1,80E+04	9,74E+03	3,38E+04
137	1,82E+04	9,98E+03	3,57E+04
138	1,83E+04	1,03E+04	3,73E+04
139	1,83E+04	1,06E+04	3,81E+04
140	1,83E+04	9,94E+03	3,57E+04
141	1,86E+04	9,48E+03	3,58E+04
142	1,90E+04	8,89E+03	3,64E+04
143	2,00E+04	8,22E+03	3,68E+04
144	2,07E+04	8,58E+03	3,76E+04
145	2,13E+04	8,97E+03	3,81E+04
146	2,18E+04	9,36E+03	3,87E+04
147	2,25E+04	1,01E+04	3,91E+04
148	2,30E+04	1,08E+04	3,92E+04
149	2,33E+04	1,15E+04	3,89E+04
150	2,30E+04	1,13E+04	3,89E+04
151	2,26E+04	1,19E+04	3,92E+04
152	2,23E+04	1,19E+04	3,89E+04
153	2,20E+04	1,18E+04	3,92E+04
154	2,14E+04	1,16E+04	4,08E+04
155	2,05E+04	1,13E+04	4,25E+04
156	1,97E+04	1,10E+04	4,35E+04
157	1,93E+04	1,07E+04	4,40E+04
158	1,92E+04	1,03E+04	4,42E+04
159	1,96E+04	1,00E+04	4,47E+04
160	1,97E+04	9,75E+03	4,56E+04

161	1,98E+04	9,57E+03	4,70E+04
162	1,97E+04	9,83E+03	4,83E+04
163	1,97E+04	1,00E+04	4,92E+04
164	1,97E+04	1,02E+04	4,92E+04
165	1,99E+04	1,04E+04	4,91E+04
166	1,97E+04	1,06E+04	4,99E+04
167	1,94E+04	1,08E+04	5,00E+04
168	1,91E+04	1,07E+04	5,01E+04
169	1,89E+04	1,10E+04	5,04E+04
170	1,85E+04	1,16E+04	5,14E+04
171	1,80E+04	1,21E+04	5,23E+04
172	1,76E+04	1,17E+04	5,26E+04
173	1,69E+04	1,12E+04	5,30E+04
174	1,77E+04	1,07E+04	5,50E+04
175	1,86E+04	1,12E+04	5,76E+04
176	1,94E+04	1,16E+04	5,99E+04
177	2,01E+04	1,10E+04	6,18E+04
178	2,06E+04	1,05E+04	6,31E+04
179	2,12E+04	9,99E+03	6,45E+04
180	2,16E+04	9,71E+03	6,38E+04
181	2,23E+04	9,40E+03	6,24E+04
182	2,23E+04	9,58E+03	6,23E+04
183	2,32E+04	1,01E+04	6,15E+04
184	2,38E+04	9,54E+03	6,05E+04
185	2,43E+04	8,95E+03	5,90E+04
186	2,46E+04	8,64E+03	5,93E+04
187	2,52E+04	8,57E+03	5,83E+04
188	2,56E+04	8,55E+03	5,73E+04
189	2,58E+04	8,51E+03	5,83E+04
190	2,67E+04	8,42E+03	5,89E+04
191	2,71E+04	8,42E+03	5,94E+04
192	2,82E+04	8,61E+03	6,16E+04
193	2,94E+04	8,81E+03	6,38E+04
194	3,05E+04	8,96E+03	6,56E+04
195	3,13E+04	9,10E+03	6,73E+04
196	3,19E+04	9,16E+03	6,83E+04
197	3,24E+04	9,20E+03	6,92E+04
198	3,25E+04	9,22E+03	6,93E+04
199	3,29E+04	9,38E+03	6,95E+04
200	3,30E+04	9,41E+03	6,94E+04
