# Supplementary material for: Recent effective population size in Eastern European plain Russians correlates with the key historical events
Source: Sci Rep. 2020 Jun 16;10:9729. doi: 10.1038/s41598-020-66734-y (PMC7298007; doi:10.1038/s41598-020-66734-y)
Supplement: Supplementary file 4 — Supplementary Information4. [file 41598_2020_66734_MOESM4_ESM.pdf]

GEN	NE	LWR-95%CI	UPR-95%CI
0	4.08E5	2.74E5	6.85E5
1	3.17E5	2.21E5	5.10E5
2	2.45E5	1.78E5	3.77E5
3	1.88E5	1.42E5	2.77E5
4	1.44E5	1.13E5	2.01E5
5	1.10E5	8.96E4	1.44E5
6	8.32E4	7.05E4	9.94E4
7	6.27E4	5.19E4	7.17E4
8	4.84E4	4.05E4	5.53E4
9	3.82E4	3.21E4	4.33E4
10	3.10E4	2.77E4	3.45E4
11	2.63E4	2.43E4	2.92E4
12	2.31E4	2.16E4	2.62E4
13	2.14E4	2.01E4	2.44E4
14	2.05E4	1.93E4	2.40E4
15	2.04E4	1.92E4	2.39E4
16	2.05E4	1.93E4	2.37E4
17	2.09E4	1.94E4	2.36E4
18	2.13E4	1.99E4	2.36E4
19	2.20E4	2.02E4	2.42E4
20	2.25E4	2.07E4	2.47E4
21	2.31E4	2.13E4	2.51E4
22	2.40E4	2.20E4	2.58E4
23	2.49E4	2.20E4	2.65E4
24	2.58E4	2.25E4	2.69E4
25	2.64E4	2.29E4	2.84E4
26	2.68E4	2.30E4	2.95E4
27	2.71E4	2.38E4	3.01E4
28	2.74E4	2.45E4	3.13E4
29	2.79E4	2.52E4	3.23E4
30	2.85E4	2.58E4	3.30E4
31	2.96E4	2.62E4	3.36E4
32	3.04E4	2.61E4	3.39E4
33	3.12E4	2.65E4	3.46E4
34	3.19E4	2.71E4	3.53E4
35	3.25E4	2.71E4	3.59E4
36	3.26E4	2.84E4	3.66E4
37	3.31E4	2.92E4	3.78E4
38	3.31E4	2.96E4	3.87E4
39	3.26E4	3.00E4	3.92E4
40	3.19E4	3.03E4	3.92E4
41	3.20E4	3.01E4	3.98E4
42	3.16E4	3.02E4	4.09E4
43	3.11E4	3.06E4	4.18E4
44	3.09E4	3.04E4	4.25E4
45	3.10E4	3.02E4	4.31E4
46	3.16E4	3.08E4	4.26E4
47	3.25E4	3.05E4	4.20E4
48	3.37E4	2.97E4	4.17E4
49	3.49E4	2.98E4	4.06E4
50	3.60E4	3.01E4	4.09E4
51	3.66E4	2.97E4	4.06E4
52	3.69E4	3.02E4	4.00E4

53	3.70E4	3.04E4	3.96E4
54	3.62E4	3.08E4	3.91E4
55	3.54E4	3.02E4	3.96E4
56	3.43E4	2.96E4	4.06E4
57	3.33E4	2.96E4	4.02E4
58	3.18E4	2.90E4	4.00E4
59	3.15E4	2.91E4	3.95E4
60	3.11E4	2.83E4	3.93E4
61	3.12E4	2.79E4	3.99E4
62	3.21E4	2.69E4	4.06E4
63	3.30E4	2.64E4	4.03E4
64	3.39E4	2.59E4	4.07E4
65	3.52E4	2.56E4	4.09E4
66	3.64E4	2.57E4	4.06E4
67	3.69E4	2.57E4	4.00E4
68	3.68E4	2.55E4	3.95E4
69	3.65E4	2.56E4	3.89E4
70	3.54E4	2.49E4	3.85E4
71	3.40E4	2.47E4	3.78E4
72	3.22E4	2.42E4	3.76E4
73	3.05E4	2.36E4	3.72E4
74	2.87E4	2.32E4	3.67E4
75	2.79E4	2.28E4	3.66E4
76	2.71E4	2.27E4	3.67E4
77	2.64E4	2.23E4	3.69E4
78	2.62E4	2.15E4	3.71E4
79	2.59E4	2.09E4	3.70E4
80	2.64E4	2.02E4	3.64E4
81	2.69E4	2.00E4	3.59E4
82	2.73E4	1.96E4	3.50E4
83	2.78E4	1.92E4	3.40E4
84	2.83E4	1.86E4	3.33E4
85	2.84E4	1.78E4	3.33E4
86	2.85E4	1.77E4	3.31E4
87	2.85E4	1.75E4	3.31E4
88	2.81E4	1.75E4	3.27E4
89	2.75E4	1.72E4	3.31E4
90	2.69E4	1.67E4	3.27E4
91	2.65E4	1.62E4	3.18E4
92	2.61E4	1.59E4	3.11E4
93	2.57E4	1.57E4	3.10E4
94	2.50E4	1.54E4	3.05E4
95	2.41E4	1.50E4	2.99E4
96	2.32E4	1.45E4	2.91E4
97	2.27E4	1.39E4	2.83E4
98	2.19E4	1.37E4	2.75E4
99	2.11E4	1.33E4	2.78E4
100	2.01E4	1.29E4	2.76E4
101	1.91E4	1.24E4	2.83E4
102	1.83E4	1.22E4	2.87E4
103	1.74E4	1.21E4	2.88E4
104	1.65E4	1.21E4	2.79E4
105	1.60E4	1.22E4	2.66E4
106	1.56E4	1.22E4	2.59E4

107	1.52E4	1.22E4	2.62E4
108	1.51E4	1.20E4	2.65E4
109	1.50E4	1.21E4	2.56E4
110	1.52E4	1.20E4	2.45E4
111	1.54E4	1.19E4	2.38E4
112	1.56E4	1.18E4	2.35E4
113	1.56E4	1.16E4	2.33E4
114	1.54E4	1.15E4	2.31E4
115	1.50E4	1.12E4	2.32E4
116	1.44E4	1.09E4	2.32E4
117	1.43E4	1.05E4	2.34E4
118	1.39E4	1.00E4	2.34E4
119	1.35E4	1.00E4	2.36E4
120	1.30E4	9.89E3	2.31E4
121	1.26E4	9.93E3	2.34E4
122	1.21E4	9.44E3	2.33E4
123	1.21E4	8.74E3	2.29E4
124	1.20E4	9.03E3	2.26E4
125	1.20E4	8.64E3	2.25E4
126	1.18E4	8.05E3	2.24E4
127	1.18E4	7.86E3	2.22E4
128	1.20E4	7.68E3	2.17E4
129	1.21E4	7.52E3	2.08E4
130	1.21E4	7.36E3	2.00E4
131	1.19E4	7.15E3	1.95E4
132	1.16E4	6.97E3	2.03E4
133	1.16E4	6.96E3	2.13E4
134	1.18E4	7.09E3	2.19E4
135	1.17E4	7.24E3	2.16E4
136	1.17E4	7.34E3	2.11E4
137	1.16E4	7.34E3	2.07E4
138	1.14E4	7.28E3	2.03E4
139	1.12E4	7.29E3	2.00E4
140	1.10E4	7.16E3	1.95E4
141	1.09E4	6.98E3	1.91E4
142	1.09E4	6.78E3	1.88E4
143	1.12E4	6.54E3	1.83E4
144	1.13E4	6.27E3	1.81E4
145	1.14E4	6.10E3	1.77E4
146	1.14E4	5.93E3	1.76E4
147	1.15E4	5.77E3	1.74E4
148	1.15E4	6.00E3	1.74E4
149	1.14E4	6.17E3	1.74E4
150	1.11E4	6.09E3	1.75E4
151	1.08E4	6.13E3	1.74E4
152	1.05E4	6.09E3	1.81E4
153	1.03E4	5.99E3	1.84E4
154	9.93E3	5.56E3	1.88E4
155	9.46E3	5.71E3	1.87E4
156	9.02E3	5.74E3	1.82E4
157	8.70E3	5.63E3	1.79E4
158	8.54E3	5.55E3	1.78E4
159	8.56E3	5.64E3	1.78E4
160	8.49E3	5.60E3	1.78E4

161	8.35E3	5.55E3	1.76E4
162	8.19E3	5.57E3	1.73E4
163	8.07E3	5.50E3	1.72E4
164	7.94E3	5.31E3	1.70E4
165	7.91E3	5.29E3	1.68E4
166	7.74E3	5.06E3	1.69E4
167	7.54E3	4.72E3	1.67E4
168	7.36E3	4.42E3	1.65E4
169	7.19E3	4.27E3	1.63E4
170	6.98E3	4.15E3	1.59E4
171	6.74E3	4.04E3	1.58E4
172	6.49E3	3.99E3	1.57E4
173	6.17E3	3.93E3	1.59E4
174	6.37E3	3.93E3	1.60E4
175	6.56E3	3.95E3	1.59E4
176	6.72E3	3.99E3	1.57E4
177	6.83E3	4.05E3	1.58E4
178	6.89E3	4.11E3	1.59E4
179	6.92E3	4.14E3	1.57E4
180	6.92E3	4.09E3	1.56E4
181	7.02E3	4.10E3	1.52E4
182	6.92E3	3.97E3	1.50E4
183	7.12E3	3.47E3	1.52E4
184	7.24E3	2.99E3	1.55E4
185	7.33E3	2.83E3	1.55E4
186	7.37E3	2.92E3	1.49E4
187	7.46E3	3.05E3	1.46E4
188	7.53E3	3.17E3	1.46E4
189	7.50E3	3.15E3	1.47E4
190	7.63E3	2.93E3	1.42E4
191	7.63E3	3.05E3	1.39E4
192	7.81E3	3.14E3	1.40E4
193	8.00E3	3.22E3	1.39E4
194	8.14E3	3.03E3	1.37E4
195	8.23E3	2.82E3	1.36E4
196	8.28E3	2.79E3	1.36E4
197	8.33E3	2.76E3	1.35E4
198	8.30E3	2.73E3	1.36E4
199	8.37E3	2.72E3	1.35E4
200	8.39E3	2.71E3	1.34E4
